# O031: Reprocessing of single-use hemodynamic catheters in cardiology-we do but how best to do it

**DOI:** 10.1186/2047-2994-2-S1-O31

**Published:** 2013-06-20

**Authors:** R Rao, R Mani

**Affiliations:** 1 Microbiology, Apollo Hospital, Jubilee Hills, Hyderabad, India

## Introduction

Hemodynamic single use devices (SUD) namely cardiac catheters are reused worldwide and in India, where cost and aggressive intervention take priority over infection prevention, is no exception. An important issue associated with the reprocessing of any SUD is the potential for subsequent transmission of infectious agents.

The reprocessing and sterilization methodology of reuse of SUD is often left to the discretion of the nursing staff (the clinicians do not play any role).The concerned staff often put the process best they know or is passed on by the predecessors. Safety, sterility issues are overlooked.

## Objectives

To evaluate reprocessing and sterilization methodology. To develop guidelines and oversee their implementations that result in standardized practices and improve the safety of hemodynamic device reuse.

## Methods

Infection Control team took over the responsibility of the process initially by a small pilot study. A robust cleaning and sterilization processes protocol based on basic microbiological principles was developed and tested for bacterial, viral and endotoxin reminants before using on patients. Initially twenty cardiac angiogram patients (consent was taken) were followed up for three months for any adverse events with reuse policy in place. Clinicians feedback was taken and modification made in the process at the end of study. It was extended it to a full fledged protocol and implemented.

## Results

This system is in place for the past 5 years and there are no adverse events reported on follow up (over 8000 angiograms so far).The facility does 12 angiogram per day and reuse is 75%.The protocol implementation has resulted in determining the number of times each catheter can be reused. The process is continuously audited and compliance to the protocol is 100%.The steps for repossessing are easy, adaptable, outcome measurable, sustainable and cost effective in resource limited settings.

## Conclusion

SUD is reused rampantly. No standard guidelines are available .The reprocessing is left to the staff who are ill informed of the consequences of reuse. The reprocessing should be based on sound microbiological and infection prevention principles and implementable .Frequent audit and follow up of patients is essential.

## Disclosure of interest

None declared.

